# A hybrid design testing a 3-step implementation model for community scale-up of an HIV prevention intervention in rural Malawi: study protocol

**DOI:** 10.1186/s12889-018-5800-3

**Published:** 2018-08-02

**Authors:** Diana L. N. Jere, Chimwemwe K. Banda, Lily C. Kumbani, Li Liu, Linda L. McCreary, Chang Gi Park, Crystal L. Patil, Kathleen F. Norr

**Affiliations:** 1 Community and Mental Health Nursing, Kamuzu College of Nursing, University of Malawi, P.O Box, 415, Blantyre, Malawi; 2 Medical-Surgical Nursing, Kamuzu College of Nursing, University of Malawi, P.O Box, 415, Blantyre, Malawi; 3 Faculty of Midwifery, Neonatal and Reproductive Health, Kamuzu College of Nursing, University of Malawi, P.O Box, 415, Blantyre, Malawi; 4 Epidemiology and Biostatistics, School of Public Health, University of Illinois at Chicago, Chicago, USA; 5 Health Systems Science, College of Nursing, University of Illinois at Chicago, 845 South Damen Ave, Chicago, IL 60612 USA; 6 Women, Children and Family Health Science, College of Nursing, University of Illinois at Chicago, 845 South Damen Ave, Chicago, IL 60612 USA

**Keywords:** HIV prevention, Implementation science, Peer group intervention, Malawi, Community implementation, Community participation, Adult, Adolescent

## Abstract

**Background:**

Scaling-up evidence-based behavior change interventions can make a major contribution to meeting the UNAIDS goal of no new HIV infections by 2030. We developed an evidence-based peer group intervention for HIV prevention and testing in Malawi that is ready for wider dissemination. Our innovative approach turns over ownership of implementation to rural communities. We adapted a 3-Step Implementation Model (prepare, roll-out and sustain) for communities to use. Using a hybrid design, we simultaneously evaluate community implementation processes and program effectiveness.

**Methods:**

Three communities in southern Malawi begin implementation in randomly-assigned order using a stepped wedge design. Our evaluation sample size of 144 adults and 144 youth per community provides sufficient power to examine primary outcomes of condom use and HIV testing. Prior to any implementation, the first participants in all three communities are recruited and complete the Wave 1 baseline survey. Waves 2–4 surveys occur after each community completes roll-out. Each community follows the model’s three steps. During Prepare, the community develops a plan and trains peer group leaders. During Roll-Out, peer leaders offer the program. During Sustain, the community makes and carries out plans to continue and expand the program and ultimately obtain local funding. We evaluate degree of implementation success (Aim 1) using the community’s benchmark scores (e.g, # of peer groups held). We assess implementation process and factors related to success (Aim 2) using repeated interviews and observations, benchmarks from Aim 1 and fidelity assessments. We assess effectiveness of the peer group intervention when delivered by communities (Aim 3) using multi-level regression models to analyze data from repeated surveys. Finally, we use mixed methods analyses of all data to assess feasibility, acceptability and sustainability (Aim 4).

**Discussion:**

The project is underway, and thus far the first communities have enthusiastically begun implementation. We have had to make several modifications along the way, such as moving from rapid-tests of STIs to symptoms screening by a nurse due to problems with test reliability and availability. If successful, results will provide a replicable evidence-based model for future community implementation of this and other health interventions.

**Trial registration:**

Clinical Trials.gov NCT02765659 Registered May 6, 2016.

## Background

Addressing HIV transmission in low-resource countries in sub-Saharan Africa is essential to meeting the UNAIDS 2030 goal of no new HIV infections [1]. Although new infections continue to decline, there were 1.8 million new infections globally in 2016, and 64% of these occurred in sub-Saharan Africa [2]. Nearly 40% of new infections occur among young people ages 15–24. Scaling-up evidence-based behavior change interventions can help prevent new infections by reducing knowledge gaps and risky sexual behaviors and increasing early testing and treatment.

Our research team developed an evidence-based behavior change intervention for HIV prevention and testing in rural Malawi, an African country hard hit by the HIV epidemic. This 7-session peer group intervention, called *Mzake ndi Mzake* (Friend-to-Friend, *Mzake*), is guided by Bandura’s social-cognitive learning theory [3] that emphasizes building self-efficacy, which has been linked with efficacy in previous meta-analyses and reviews [4–9]. Each session incorporates games and role-plays that rehearse specific steps needed for HIV prevention, such as discussing condom use with a partner. Importantly, the intervention is designed for all reproductive aged individuals regardless of age or gender. Using a quasi-experimental design, we compared a random sample of adults and youth in communities that received *Mzake* and control communities. People in the *Mzake* communities reported significantly more HIV prevention behaviors, including more condom use (Adults, 25% vs. 12%; Youth, 70% vs. 49%) and more HIV tests within the past year (Adults, 18% vs. 10%; Youth, 14% vs. 7%) [10–12].

With efficacy established, this intervention is ready for wider dissemination to other rural communities in Malawi. In the efficacy study, volunteer health workers served as peer leaders and provided training. However, persistent health worker shortages mean that Malawi’s health system does not have the capacity to lead community scale-up because they must meet expanding demands for HIV testing and treatment as well as other health needs.

We opted to use a promising alternative to reach rural communities – community ownership and leadership of scale-up of this evidence-based HIV prevention intervention. This approach falls within the broad framework of community-based participatory research (CBPR), where the agenda is set collaboratively by the community and researchers [13–17]. Numerous studies have documented that a participatory approach integrating the perspectives and interests of community leaders and members, researchers and other stakeholders enhances effectiveness and sustainability [18]. We chose to bring the intervention to Phalombe district in southern Malawi, where the need for HIV prevention is especially great. HIV prevalence in the Southern Region is 16%, twice that of other regions in Malawi (MPHIA 2016) [19], and Phalombe is a low-resource, rural district that has received few HIV prevention initiatives.

### Preliminary work prior to funding

The first step in testing this CBPR alternative was to determine if local communities were ready to take up the challenge and identify what they would need to do so. We met with local leaders and community members, described the intervention, and discussed their willingness to take on *Mzake*’s implementation. All were enthusiastic about introducing HIV prevention in their communities. One leader observed that the HIV epidemic continues to hurt families and said, *“None of us are spared from the agony of losing many loved ones …. We would do anything possible to support the study … for everyone to benefit from it.”* Leaders noted that they could use same strategies and resources they now use to organize local traditions and church or volunteer activities but would need to acquire appropriate health knowledge and program-specific implementation skills. One leader observed, *“We have heard about prevention messages on the radio, in meetings, through drama and on the national day for HIV prevention. But when we go home we really do not understand how we can get our people protected.”* These community discussions led to the development of a local multi-sectoral partnership with communities at the center that ensured their ongoing access to local support and resources. The District Commissioner (head of district government) agreed to integrate this program into their district agenda and resource allocation. The district’s health team agreed to contribute health knowledge, local statistics and training support. The research team agreed to provide guidance for implementation and *Mzake* program-specific knowledge. Traditional leaders at all levels agreed to help mobilize communities and disseminate progress.

To move forward, we needed a simple but effective implementation model that communities could easily use as they took ownership of the *Mzake* program and rolled it out. Most implementation models are developed for use by researchers and health professionals in clinical settings [20]. These were too complex and abstract for community use. The model needed to be accessible to individuals with less education and little exposure to formal research. An evidence-based model originally used to implement a hospital-based intervention in South Africa was the most appropriate because it had been used successfully in Africa with clinical staff, some of whom had relatively low education [21–24]. In consultation with Dr. Bergh, one of the model’s original developers, we adapted this model for community use. We simplified it by reducing the model from six to three major steps: *prepare, roll out and sustain.* We also developed *Mzake*-specific benchmarks for each step; the benchmarks guide implementation and are also used by communities to document their progress.

The purpose of this research is to test whether three rural communities in Phalombe, Malawi can successfully implement the *Mzake* intervention using our simple 3-Step Implementation Model. The specific aims are to:

1. Prepare and support three communities as they use the 3-Step Model to implement the *Mzake* peer group intervention to reduce new HIV infections.
2. Identify implementation patterns across sites and over time using mixed-methods analyses.
3. Assess *Mzake* effectiveness when implemented by communities.
  - Hypothesis: At each wave, participants who have completed the program will have significant improvement in HIV-related behavioral outcomes and sexually transmitted infections (STIs) compared to those in communities where the intervention has not yet been introduced.
4. Evaluate whether the 3-Step Model is feasible, acceptable, effective and sustainable when used by communities to implement an evidence-based intervention.

Results of this study will make a major contribution to health promotion policy and to implementation science. If communities can take the lead in scaling-up evidence-based interventions to reduce new HIV infections, this will offer a new approach to scaling up HIV prevention without overburdening the health system. If our simple 3-Step Model helps community members achieve this, the model and lessons learned about its use in three different communities will make important empirically validated contributions to implementation science.

## Methods

### Design

This study uses a hybrid design, which was developed to advance implementation science by examining both implementation processes and effectiveness in a single study [25, 26]. We give equal attention to both processes and effectiveness evaluation, a balanced design recommended where there is already evidence supporting efficacy [25].

**Fig. 1:**
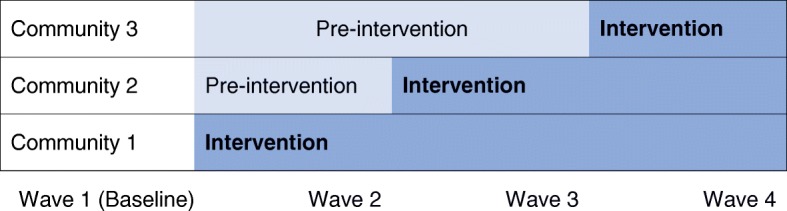
Stepped wedge cluster randomized design with evaluations at Waves 1-4

We use a stepped wedge design to evaluate effectiveness, with staggered introduction of the intervention in three communities in randomly assigned order (Fig. 1). The stepped wedge design [27–29] has the advantage that all groups receive the intervention, which is ethically important, especially in a community intervention. This design is efficient because the repeated measures allow participants to serve as controls until the intervention is introduced in their community. In each community, the first 144 adults and 144 youth are asked to both receive the intervention and to participate in the effectiveness evaluation. Outcome measures are assessed at baseline and after each community rolls out the intervention.

### Setting and sample

The study takes place in Phalombe District in southern Malawi. The district has a population of nearly 350,000 [30]. In consultation with District leaders, we identified one sub-district area as the most appropriate place to test the 3-step model. This area is the largest in the district; a third of the district’s population resides there. The leaders in that area selected three communities as sites for implementation.

The sample includes both the implementation partners described above, who share their perspectives on the implementation process (Aim 2), and community members, who participate in the intervention (Aim 1) and its effectiveness evaluation (Aim 3). District level leaders from government, the health sector and traditional leaders collaborate for implementation and are included in qualitative evaluation. Implementation partners in each of the three communities include a coordinating committee that will oversee implementation and the trained peer leaders. Eligibility criteria for implementation partners include being in the designated role and willingness to participate.

The community member participants include adults and youth (ages 13–19) who take part in the 7-session peer group intervention. The first 144 adults and 144 youth in each community participate in both the peer group intervention and the repeated effectiveness surveys. Participants also provide information for the process evaluation during periodic group session observations, focus groups and repeated individual interviews. Eligibility criteria for community participants include residing in the designated community and, for youth, being within the designated age range and having parental permission to participate if under age 18. We subsequently increased the number of youth recruited, because they are more likely to move away over time.

#### Power analysis, Aim 2 effectiveness

We performed a power analysis for the stepped wedge effectiveness component to determine sample size. Conventional power calculation programs are not able to consider both the intra-class correlation (ICC) for the intervention clusters, and the ICC for repeated measures of individuals over time. Therefore, we performed 1000 simulations using the stepped wedge design, for both continuous outcomes (analyzed using mixed-effects regression) and binary outcome (analyzed using mixed-effects logistic regression) [31, 32]. We conservatively set cluster-level ICC to be 0.10, based on two previous community or classroom studies that found an ICC of 0.05 [33, 34]. Individual-level ICC was set to range from 0.2 to 0.4, as is typical for correlations between repeated measurements of an individual. We set retention rates at 85, 80, and 75% at waves 2 through 4, a conservative estimate given our prior study with 80% retention at 18 months. Expected effect sizes (0.10 to 0.20 for continuous outcomes; odds ratios ranging from 1.2 to 2.2 for dichotomous outcomes) were based on significant outcomes in our previous efficacy study [10, 11] or, for comprehensive HIV knowledge and sexually transmitted infection (STI) prevalence, a 2010 national survey [35]. With 80% statistical power, the sample of 432 persons (youth or adults) with retention rates of 75% at the last wave, supported a minimum detectable effect size of 0.13 for continuous outcome variables, and a minimum detectable odds ratio of size 1.36. Thus, we have sufficient power to detect all expected intervention effects for outcome variables for adults and youth separately.

### The intervention

Community leaders are oriented to the 3-Step Model by the research team and establish a coordinating committee to oversee implementation of the *Mzake* intervention using the 3-Step Model. We use the procedures and materials that were developed for the *Mzake* efficacy study. Leader training is highly interactive with extensive practice and feedback so that leaders learn how to facilitate the group rather than lecturing, which is the usual learning modality [36, 37]. Because the capacity to train new leaders needs to be established locally, we plan to establish a group of health worker volunteers who can serve as trainers. Interactive assistance is provided during implementation of *Mzake* using the Model. This assistance is designed to gradually decrease as communities build capacity for independent implementation. To support independence, each site also receives a Toolkit which includes the Coordinating Committee Implementation Guide, Benchmarks Checklist, the Peer Leader Training Guide and the Adult and Youth *Mzake ndi Mzake* Manuals, and the physical materials required for *Mzake* sessions. During the pilot we made a training video showing how to facilitate a group using examples from actual group sessions. Prior research has found that implementation proceeds more quickly with technical assistance, although the Toolkit alone may be enough for some sites [24].

### Measures

This mixed-methods evaluation uses multiple types of data from different sources. Table 1 below summarizes the data sources and measures. Most materials are already translated, but new materials are translated using a team approach throughout the study. After initial translations and back-translations, a translation team (bilingual nurse, academic, and lay persons) reach consensus regarding differences, ensuring that the materials are accurate, comprehensible and conceptually equivalent [38–41].

**Table 1:** Evaluation measures

Aim	Measures
1. Implementation progress	
Benchmarks	26 yes/no items (1 point for each done) measuring implementation progress at each site. Numeric summaries are also recorded for peer leaders trained, 7-session peer groups and boosters held (# completed, # of adult and youth participants). Scored every 6 months. Summaries for each community and the total are presented to the community and district leaders and integrated with related local health outcome data (e.g., # of HIV tests provided, % of pregnant women tested) routinely collected and provided by the District Health Officer.
2. Implementation patterns and process	
Peer group fidelity ratings	• Structured fidelity ratings by the Research team (trained to an inter-rater reliability of 0.85) [37]. At least one of the early sessions of all peer leaders plus randomly selected groups thereafter is observed. • Peer leaders’ self-assessments and attendance sheets
Observation notes on meetings	Study notes by the research team (semi-structured observations of all meetings attended) documenting type of meeting or observation, date, site, and discussion related to successes, challenges, if problem was solved; and personal interactions
Annual focus group & Individual Interviews	7–12 focus group interviews with district and community implementation partners and 8–12 individual interviews with implementation partners including local “champions” and unsupportive individuals. Coded to identify successes, problems/barriers/ solutions, and appraisal of support and technical assistance
3. Effectiveness for Participants	
Survey	• Demographics (e.g., Age; education; current partner status) • Program time/dose: Date of last session attended; # of sessions attended • Comprehensive HIV-related Knowledge: (5 HIV & 4 PMTCT items [35]) • Safer sex Practices over the last 2 months [10] (Primary Outcome) (a) *Self-efficacy scale* (4 items, # discussed: HIV prevention, condom use, being faithful, HIV test with partner; responses very, somewhat, not confident, range 3–12); (b) Partner communication index (4 yes/no items, # discussed: HIV prevention, condom use, being faithful, HIV test with partner); (c) If abstained; d) If sexually active, condom use (always, sometimes, never); • Sexual Risk Index [10] (had unprotected sex, more than one partner, sex for money, sex while drinking, sex with a stranger; 5 items, # Yes) • If used a condom at last sexual relations [35] • If not sexually active at baseline, whether sexual debut occurred (youth only) • HIV Test (Primary Outcome): If had HIV test in last year, yes-no; if yes, whether tested with partner [10]
STIs	• Original Plan: Tests for clinical and sub-clinical gonorrhea, chlamydia, and syphilis using minimally invasive rapid tests appropriate for use in a community setting without electricity or access to laboratory or examination facilities. This plan had to be modified because tests were withdrawn from the market and/or found to be unreliable in new research [45, 46]. • Revised Plan: Detailed interview regarding STI symptoms experienced in the last week and in last 6 months (with referral for clinic follow-up when indicated)
4. Integration of patterns with effectiveness and sustainability	
All above data	Within and cross-case matrices: implementation patterns and relationship to progress benchmarks, effectiveness, if sustained program

### Implementation procedures

#### District-level preparation (months 1–8)

We first obtain ethical approval at both universities, hire staff, establish an office and train the research team, including training or updating in ethical conduct of research and a confidentiality pledge. We then reconfirm our collaboration with district partners and community leaders, make plans and orient them to the intervention. The district nursing officer identifies up to 12 health workers who attend a Training of Trainers workshop led by the Co-Investigators. Training ends with a pilot, where health workers practice conducting peer groups with community volunteers. Sessions are videotaped and used to develop a training video to illustrate effective group facilitation techniques.

#### Implementation at 3 sites

We test the 3-Step Model in the 3 sites sequentially, with the order of introduction at each site randomly assigned during a public meeting. The same sequence of implementation steps and activities occurs at all sites.

During the *Prepare* step (2 months), community leaders are oriented to the program and form a site coordinating committee comprised of respected adults active in community affairs. The committee is oriented to the 3-Step Model and *Mzake.* They learn about the benchmarks which they use to develop a plan and evaluate progress every 6 months (Table 1). After making a community implementation plan, they recruit volunteers to become peer leaders. The volunteers are trained to deliver *Mzake,* maintain group records and complete a brief self-evaluation of fidelity. *The primary capacities built during this period are conceptual understanding of the* 3-Step Model *and Mzake; planning and coordination for the coordinating committee and peer group leadership skills for peer leaders.*

During *Roll-Out* (8 months), *Mzake* is introduced to community members at village meetings and those who are interested sign up to be in the peer groups and complete informed consent. The first 144 participants must also agree to complete the repeated effectiveness surveys. Peer leaders conduct the first round of 12 groups with adults, then reflect on their experiences with the coordinating committee and make any modifications. Because talking about sexuality with youth is a sensitive topic in Malawi, the leaders obtain experience and confidence with adult groups and then the same peer leaders offer 12 groups with youth. During both adult and youth groups, the research team rates fidelity and provides supportive coaching for each pair of leaders during at least one early session and one later session, rating fidelity and providing coaching to the leaders. After these initial peer groups are completed, the peer leaders and committee will meet to reflect on lessons learned and prepare for future implementation. The lessons learned from both rounds of groups will be shared with implementation partners and iteratively used to modify the 3-Step Model. *The primary capacities built during this period are: experience in using the* 3-Step Model *and offering Mzake, practical logistics for local implementation and completing the benchmarks self-evaluation for committee members.*

*Sustaining* is divided into two periods, 11 months of sustaining with partial support, followed by independent sustaining up to the last year of the grant period. Because of the staggered implementation plan, Site 1 will have 30 months of independent sustaining, Site 2 will have 18 months and Site 3 will have 8 months of independent sustaining. During sustaining with support, sites are told that they will receive a specific amount of money for 11 months of sustaining activities. The committee members receive advanced training regarding sustaining and discuss their vision for what should happen. They receive additional guidance on planning activities, budgeting for them and accounting for expenditures. Activities during sustaining include training more peer group leaders and possibly new committee members, holding *Mzake* groups for new participants and in new areas, and booster activities for those who have already attended *Mzake*. One important part of sustaining is ensuring the capacity to train new peer group leaders, and the committee may decide to ask for training of interested peer leaders as trainers. They then decide how to allocate their efforts and carry out their plans, monitoring what is happening and making any needed adjustments. Toward the end of sustaining with support, the committee learns how to write a proposal and budget in a workshop and seeks funding from us and other sources. Our staff is available but does not initiate regular contacts as we do during roll-out.

During independent sustaining, the community should begin to expand to new areas and obtain new support for their work. They can apply to us for a small grant and to the District Commissioner’s office and other funders for additional months of partial funding. The Study Team continues “hands-off” observation of progress during the remainder of this period [23]. The intention is for each community to continue indefinitely. However, we only observe progress up to Month 52. *The primary capacities built during this period are independently training new peer leaders, conducting booster sessions, expanding to new parts of their own site and possibly to other sites, maintaining financial records, and preparing proposals and budgets to sustain activities.*

### Evaluation procedures

#### Aim 1 implementation progress

Data includes benchmark scores rated by the site coordinating committee every 6 months, with results reported to community leaders and other implementation partners. Summary benchmark indicators can be displayed using simple bar-graphs and maps, so patterns can be easily recognized by community members, implementation partners, and researchers. The benchmark information from all three sites can be summarized and tracked. Health centre data will be used to track changes over time in the number of condoms distributed, HIV tests requested and related services provided in clinics serving the three communities.

#### Aim 2 implementation patterns

Patterns will be assessed using qualitative data from our study team’s observations, notes of meetings they attend, and annual individual and focus group interviews. Interviews will include persons who have been either highly enthusiastic (e.g., “champion” in the District Advisory Committee, an especially “productive” peer leader) or unenthusiastic (e.g., person who expressed reservations about the peer group contents, peer leader who discontinued participation). Quantitative measures include the benchmarks described above and independent evaluations of Mzake fidelity by the research team.

#### Aim 3 effectiveness measures

Following the stepped wedge design [28, 42, 43], all adult and youth participants are recruited and consented at Wave 1 (baseline). The data collection procedure is the same for each wave. Each participant completes a brief Audio Computer-Assisted Self-Interviewing (ACASI) survey four times (Waves 1–4). We used ACASI in a previous study with young women with no problems. ACASI promotes privacy and higher reporting of sensitive sexual behaviors [44]. and was well-received by participants. To retain participants in the repeated surveys, we use standard retention strategies, including respectful interactions, repeated telephone contacts when necessary, refreshments and a small cash allowance for travel and associated.

We originally planned to obtain STI biomarkers (as indicators of unprotected sex) using rapid tests to assess the presence of three prevalent sexually transmitted infections (STIs; gonorrhea, chlamydia, and syphilis) at two time points. However, several recent studies found that the non-invasive rapid tests for gonorrhea and chlamydia had unacceptably low reliability and one was withdrawn from the market [45, 46]. Instead, we substituted a detailed systematic STI symptoms interview recommended by Dr. Supriya Mehta. In her Kenya research, she found that interviews by a nurse were accurate in identifying who had an STI based on laboratory follow-up (personal communication, 11/23/2015). The nurse will refer anyone with current symptoms and follow-up within a week to ensure compliance. We discussed the planned protocol modification with our program officer at the National Institutes of Health and obtained approval. We then modified the protocol in clinicaltrials.gov.

#### Aim 4 integration

Integration of implementation patterns with effectiveness and sustainability will use all of the above data. No new data are collected for this aim.

### Analysis

#### Data management and iterative analysis

Throughout the study, analysis is continuous and iterative, integrating new data as they become available. Data management protocols promote proper and timely preparation of data for analysis and secure data storage. All data are identified by location and date. A unique individual code is assigned to Individual-level data, including effectiveness evaluation data and individual interviews. Master lists linking names and code numbers will be securely stored separately from the data. Only de-identified data will be transferred from Malawi to UIC using a secure transfer system. *Quantitative data* collected include the benchmarks, Effectiveness Evaluation surveys and STI tests. The ACASI survey data are automatically entered into a database. All other data are entered by data clerks with 100% verification and transferred to data analysis programs (SAS, SPSS). *Qualitative data* (hand-written study notes and audio-recorded focus groups and individual interviews) are processed following Huberman and Miles’ recommended strategies to code, store, and retrieve data [47]. Recorded interviews and hand written notes are typed from notes or transcribed verbatim, referring to the original recordings to verify accuracy of the typed transcripts. Materials in Chichewa are translated using the same translation process described above in Measures. Transcripts and notes are imported into a qualitative data management and coding program that accepts quantitative as well as narrative data.

#### Aim 1 (implementation progress)

We examine whether each site is using the 3-Step Model to implement the program and the extent and fidelity of implementation. We will analyse progress through changes in the benchmark scores across time within each site. We then examine the similarities and differences in progress across sites. Fidelity to the model is assessed by examining whether the steps of the 3-Step Model are being followed in the recommended order, adapted or skipped (e.g., variation in steps). *When Aim 1 analysis is complete, we will have a description of how implementation has unfolded at each site over time and across sites, including which sites continued implementation, amount of implementation, and degree of fidelity to the 3-Step Model.*

#### Aim 2 (implementation patterns)

We use mixed methods analysis to examine the patterns that emerge over time and across sites and the contextual factors that influence these patterns. Qualitative data are coded based on the conceptual frameworks that underlie the 3-Step Model and *Mzake* [21, 36]. We focus on reported successes, challenges, solutions tried and whether successful, as well as champions and naysayers who emerge. We will be sensitive to new discoveries that emerge, which will be developed into new codes. Final codes are compiled in the master codebook and applied to the coding of all qualitative date. The data will be double-coded until intercoder reliability exceeds 85%. Thereafter, 20% of randomly selected materials will be double-coded.

We use quantitative descriptive statistics for initial analysis of the benchmarks and fidelity assessments [22, 37]. The benchmarks provide a way to assess fidelity to the 3-Step Model and contextual adaptation, as described above (Aim 1). Fidelity to *Mzake* is assessed using a simple rating by the peer leader. During this implementation study, the peer leader self-ratings will be validated using periodic independent detailed observation by the research team. If the peer leader self-ratings correlate with our observations, self-ratings can be used by communities to monitor fidelity independently. Comparing fidelity scores with those found in the *Mzake* efficacy study provides a standard for the level of fidelity sufficient to produce the desired outcomes. We will compute the fidelity score for each group observed. We will then create three mean fidelity scores at each site, overall fidelity, early fidelity (during roll-out) and later fidelity. We expect peer leader early fidelity to be acceptably high and to increase over time as sites and peer leaders gain experience. We will also describe variations by peer group gender and age category (youth or adult). For each site, we will annually produce a case summary including both qualitative and quantitative data summarizing processes and contextual factors affecting implementation.

Finally we integrate these different data using well-established mixed methods analyses as described by Cresswell [48] and Greene [49], in which both qualitative and quantitative data are transformed into comparable categorical or interval-level data. These data are then brought together using within and across case matrices that allow identification of co-occurrences, patterns and variations [47, 48, 50]. Throughout the mixed methods analysis, we will observe the ways in which communities balance fidelity to the core components of the intervention and adaptations that make the intervention work for their context. *At the conclusion of the analysis for Aim 2, we will identify the major implementation patterns and consistency and variation across sites and time.*

#### Aim 3 (effectiveness analysis)

The effectiveness of the *Mzake* intervention when offered by communities is determined by survey data and STI symptom interviews with a nurse at Waves 1–4. We will complete a CONSORT diagram showing recruitment, retention and loss to follow-up at each wave. We then examine significant factors associated with lost to follow-up. Identified attrition factors will be controlled in later analyses.

To evaluate the effectiveness of the intervention in improving HIV-related outcomes, we will use multi-level mixed-effects regression (for continuous outcomes) and logistic regression (for dichotomous variables) models [51]. These methods allow us to take into account time of implementation as well as lack of independence among repeated measurements and members of the same peer group. The hierarchical structure of the data is conceptualized as repeated outcome measures (level 1), nested within individuals (level 2) and nested within peer groups (level 3). Random individual and peer group effects will account for correlations between repeated measurements and between individuals from the same peer group. Intervention condition will be a time-varying covariate that reflects the stepped-wedge design in which individuals receive intervention at different stage of the study. Site and demographic factors (both individual level and community level) will be treated as fixed-effects covariates to account for differences. Time trend interactions for intervention and imporant factors will be tested to identify differential effects over time. Youth and adult data will be analyzed both together and separately. Model selections for the number of random effects and the variance-covariance structures for the outcome variables will be performed using likelihood ratio tests. Backward selection will be employed for the fixed effects. *Aim 3 analysis identifies effectiveness overall and by site.*

#### Aim 4 (integrated 3-Step Model analysis)

The 3-Step Model’s feasibility, acceptability, effectiveness and sustainability for implementation by communities is evaluated by integrating data and analysis results from Aims 1–3. Our analysis focuses primarily on the relationships among the extent and patterns of implementation identified in Aim 2 and whether implementation variations are related to effectiveness and implementation success and sustainability. This mixed-methods analysis can be conceptualized as a comparative case study with 3 cases [49, 52]. We use the same mixed-methods approach and methods described above. We first develop within and across case matrices to identify patterns and relationships, and then examine detailed case summaries to understand the complex context that surrounds similarities and differences. For example, if only 2 sites were able to use the 3-Step Model with fidelity to sustain implementation to the end of the study period and both had high effectiveness, a close examination of the less successful site should identify potentially important differences and point to aspects of the 3-Step Model that need improvement. *Aim 4 analyses will link the community implementation process and patterns of using the 3-Step Model with the effectiveness evaluation results and sustaining of the intervention. Lessons learned from these analyses will tell us how well communities were able to use this 3-Step Model and help identify contextual factors that appear to enhance or inhibit sustainability.*

## Discussion

This study will test whether community members can take ownership and successfully scale-up implementation of an evidence-based intervention. While many community studies use a participatory approach, there are few published accounts where communities organize and manage implementation. Our hybrid design enables us to simultaneously examine facilitators and challenges affecting the community implementation process and program effectiveness when implemented by community members under real-world conditions.

Two of the three communities have already begun rolling-out the intervention. Leaders have established a coordinating structure and a plan for spreading the intervention in their villages. Our district-level partners have made the contributions they agreed upon, although communities have not yet reached the point of seeking additional local funding for long-term sustainability.

Along the way, we have encountered several challenges. Initiating a complex project in two linked but geographically distant universities is never simple. Executing a subcontract and transferring funds, and then completing local hiring, setting up an office in the community and team training took many months. Consequently, although we continued planning with district and community leaders, project activities at the community level were delayed. These delays were particularly frustrating for community leaders and members, who had been waiting from the time we met with them as we developed our NIH proposal. Keeping community partners engaged from initial project planning through funding and then project set-up is a major challenge, yet bringing them into early stages of project development is an essential part of a participatory approach. We attempted to manage this through frequent contacts and transparency about delays, but this issue remains problematic in participatory research and university-community collaboration.

We also needed to make two modifications in the protocol during early implementation. As described above, shortly after the project began, new data identified that the non-invasive rapid STI measures that had been deemed appropriate for community use had unacceptably low reliability. Therefore, we transitioned to an STI symptoms interview by a nurse. The other change was the addition of a pilot with youth. We initially planned to pilot the program only with adults, but after working with peer group leaders we realized that they lacked confidence about offering *Mzake* to youth. Therefore, we conducted a pilot with youth, which gave the peer group leaders confidence and a better understanding of the need to discuss sexuality issues with youth openly. Lessons learned in the pilot also led our team to create a separate session guide with age-appropriate content and role-play scenarios with more participants to enhance engagement with youth.

This study provides a new approach for scaling-up effective interventions to reduce new HIV infections and will have a major impact on implementation science, especially for community-led implementation. Community-wide implementation of an efficacious intervention has high potential to change sexual risk and HIV testing behaviors, and thus reduce new HIV infections, without imposing new burdens on the health system. The 3-Step Community Implementation Model fills an important gap in implementation science. Moreover, the model is potentially generalizable to community scale-up for a wide variety of evidence-based health interventions. Lessons learned regarding implementation similarities and differences across three different communities and effective strategies to support community scale-up will advance implementation science.

## Abbreviations

AIDS: Acquired immunodeficiency syndrome
HIV: Human immunodeficiency virus
ICC: Intra-class correlation
STI: Sexually transmitted infection
